# Apelin protects against ischemia-reperfusion injury in diabetic myocardium via inhibiting apoptosis and oxidative stress through PI3K and p38-MAPK signaling pathways

**DOI:** 10.18632/aging.104106

**Published:** 2020-12-20

**Authors:** Songtao An, Xi Wang, Huairui, Shi, Xueqiang Zhang, Hua Meng, Wenbo Li, Dongchang Chen, Junbo Ge

**Affiliations:** 1 Department of Cardiology, Henan Province People's Hospital, People's Hospital of Zhengzhou University, Zhengzhou 450003, Henan, China; 2 Department of Cardiology, Zhongshan Hospital, Fudan University, Shanghai 200032, China; 3 Department of Cardiology, Hongxing Hospital, Hami 839000, Xinjiang, China

**Keywords:** diabetic myocardial ischemia reperfusion injury, ischemia and type 2 diabetic mellitus, apelin, oxidative stress

## Abstract

Among all diabetes mellitus-associated cardiovascular diseases, morbidity of diabetic myocardium with ischemia reperfusion injury (D-IRI) is increasing year by year. We aimed to discover a therapeutic biomarker and investigate its mechanism in D-IRI. High-fat diet and streptozotocin-induced diabetes rats were operated with IRI or sham. Recombined lentiviral vector encoding Apelin was injected into D-IRI rat via tail vein. Cardiac function, infarct size, cellular death and oxidative stress were major outcome measures. Cardiomyocyte ischemia reperfusion injury was more serious in D-IRI rats than in non-diabetes ischemia reperfusion injury (ND-IRI) rats. The secretion of NTproBNP was increased in D-IRI compared with ND-IRI. Bcl-2 expression was decreased, and Bax and cleaved caspase-3 expression was increased in D-IRI rats compared with ND-IRI rats, which were reversed after treatment with Apelin. Apelin-upregulation improved cardiomyocyte ischemia reperfusion injury and decreased NT-proBNP levels in D-IRI rats. Apelin overexpression enhanced PI3K and eNOS levels while reduced those of p38-MAPK and iNOS in D-IRI rats. Apelin overexpression protected against D-IRI through inhibiting apoptosis and oxidative stress via PI3K and p38MAPK signaling pathways in D-IRI rats. These findings provide critical new insight into understanding of Apelin's cardio-protective effects, which may become a novel therapeutic target for the diabetic IRI patients.

## INTRODUCTION

Diabetes mellitus (DM) is a metabolic syndrome. Epidemiological discoveries show that the DM is the most vital risk factor for the myocardial, the cardiovascular disorders, and the ischemia reperfusion injury (IRI), and the mortality in these diseases is 2 to 6 times higher in the DM patients compared to the non-DM patients [1]. Yin, et al. found that the poor prognosis of DM is at least partly correlated with the endogenous cardio-protective strategies in DM, for example, the ischemia reperfusion injuries (IRI) [2]. IRI mainly occurs an acute ST-segment elevation myocardial infarction in patients who have experienced either thrombolytic therapy or primary percutaneous coronary intervention (PPCI) for reducing acute myocardial ischemic injury and limiting the size of myocardial infarction. However, the myocardial reperfusion always evokes myocardial reperfusion injury [3, 4]. Therefore, we believed that establishment of an effective strategy decreasing the degree of infarction in the process of IRI is pretty significant in clinical cases.

The chaos of renal-angiotensin system (RAS) is associated with initiation of cardiovascular disorder [5]. Angiotensin II (Ang II), the main regulator of RAS, regulates and activates Angiotensin II type 1 (AT1) and the reduced form of NADPH oxidase to trigger superoxide anion. Additionally, NO and oxidize tetrahydrobiopterin (BH_4_) can interact with superoxide anion and peroxynitrite. The endothelial nitric oxide synthase (eNOS) leads to uncoupling and then endothelial dysfunction-associated oxidative stress is emerged [6–8]. Apelin was firstly discovered from bovine stomach extracts in 1998, which is a novel adipocytokine secreted by the white adipose tissues and could bind to the Apelin receptor (APJ, orphan G protein-coupled receptor) with high affinity [9]. Apelin, activated under cardiovascular injury or disorder, is a homologous molecule of Ang II and belongs to the RAS [10, 11]. Gurzu et al. reported that the Apelin restrains the Ang II-mediated vasoconstriction via binding to the Apelin receptor (APJ) [12]. The RAS regulates the cardiovascular system, kidney system, and blood pressure [13]. Apelin is distributed in several tissues and plays multiple bioactivities via combination with APJ through autocrine, paracrine and endocrine pathways [14–16]. The decrease of arterial pressure induced by Apelin is largely attributed to the activation of eNOS [17]. The angiotensin converting enzyme 2 (ACE2) is a negative modulator of the RAS, and Apelin knock-down could suppress the expression of ACE2 [18, 19]. The Apelin signaling system is implicated in various physiological effects and participates in multiple pathological processes, especially in the cardiovascular disorders (such as heart failure, hypertension, myocardial injury, atherosclerosis), diabetes complicated with microvascular diseases, tumors, ischemia reperfusion injuries, as well as the pre-eclampsia [20]. Large number of studies indicated that increased angiogenesis offers oxygen and nutrients to ischemia area of myocardium. W. Wang, et al. and S. Kumagai, et al. published that Apelin promotes angiogenesis in myocardial infarction (MI) [21, 22]. Furthermore, Apelin-13 enhances the expression of vascular endothelial growth factor (VEGF), VEGF receptor-2 (VEGFR-2) and eNOS, enhancing the capillary density in the heart of MI rat [23].

However, there are also some un-resolved problems in the diabetic myocardium with ischemia reperfusion injury (D-IRI) injury. Firstly, specific mechanism of the D-IRI has not been fully clarified. Secondly, effectively therapeutic strategies for D-IRI still need better investigation. Therefore, the present study utilized D-IRI rat model to explore potential therapeutic strategy and related mechanisms of Apelin in D-IRI injury.

## RESULTS

### Establishment of animal models

In T2DM rat model, rats were successfully established as DM. Twenty-four of thirty-seven rats were established as DM complicated with IRI (D-IRI), and six of eight rats were established as diabetic control (D-sham). Twenty-four of thirty rats were established as IRI (ND-IRI), and seven of eight rats were established as control (ND-sham).

### Cardiac function was significantly injured in D-IRI rats

The cardiac function of rats in different groups was evaluated by ultrasonic inspection the 6^th^ week after IRI surgery. The results indicated that the LVIDd, LVIDs and the LVPWs were increased significantly, while the EF and FS were decreased significantly in the ND-IRI and D-IRI groups compared to ND-Sham and D-Sham groups, respectively (Table 1). Besides, LVIDd, LVIDs, LVPWs were increased markedly and the EF and FS were decreased markedly in D-IRI group compared to ND-IRI group (Table 1, P<0.01). Eight weeks after surgery, the hemodynamic parameters were evaluated (Table 2). The results displayed that the LVEDP was increased distinctly while LVSP and LVDP/dtmax were decreased distinctly in the ND-IRI and D-IRI groups compared to ND-Sham and D-Sham groups, respectively. Besides, the LVEDP was increased but LVSP and LVDP/dtmax were decreased significantly in D-IRI group compared to ND-IRI group. Furthermore, significantly increased LVEDP as well as decreased LVSP and LVDP/dtmax were observed in D-Sham group compared to ND-Sham group. NT-proBNP is a marker of myocardial ischemia. The concentration of NT-proBNP in D-IRI and ND-IRI group was obviously promoted compared to D-Sham and ND-Sham groups, respectively. The concentration of NT-proBNP in D-IRI group was also promoted significantly relative to ND-IRI group. Moreover, the NT-proBNP was obviously enhanced in D-Sham group compared to ND-Sham group. In conclusion, rats with diabetes had bad cardiac function and myocardial ischemia/reperfusion aggravated these dysfunctions.

**Table 1:** Ultrasonic inspection results for rats.

**Groups**	**ND-Sham (n=8)**	**ND-IRI (n=13)**	**D-IRI (n=14)**	**D-Sham (n=7)**
LVIDd (mm)	6.45±0.38	10.21±0.67^*^	15.32±0.82^*$^	9.35±0.53^#^
LVIDs (mm)	2.98±0.57	6.16±0.38^*^	8.89±0.98^*$^	4.02±0.68^#^
EF (%)	67.58±4.69	50.73±5.36^*^	35.73±2.88^*$^	56.58±3.22^#^
FS (%)	50.13±6.31	29.82±6.21^*^	26.82±4.39^*^	40.13±4.35^#^
IVSd (mm)	1.04±0.25	1.33±0.59^*^	1.83±0.59^*$^	1.44±0.45^#^
IVSs (mm)	1.76±0.56	1.98±0.47	3.02±0.63	2.06±0.55^#^
LVPWd (mm)	1.73±0.26	1.99±0.17^*^	2.15±0.17	1.92±0.26^#^
LVPWs (mm)	1.58±0.25	2.28±0.10^*^	3.07±0.12^*^	2.47±0.35^#^

Data are presented as mean ± standard deviation. ^*^P<0.05 represents the values compared to ND-Sham group. ^#^P<0.05 represents the values compared to the ND-sham group. ^$^P<0.05 represents the values compared to ND-IRI group. ND-Sham: Non-diabetes sham group; ND-IRI: Non-diabetes ischemia reperfusion injury; D-IRI: Diabetes ischemia reperfusion injury; D-Sham: Diabetes sham group.

**Table 2:** Cardiac hemodynamic in non-diabetic or diabetic rats with or without ischemia reperfusion.

**Group**	**ND-Sham (n=8)**	**ND-IRI (n=13)**	**D-IRI (n=14)**	**D-Sham (n=7)**
LVEDP (mmHg)^a^	3.76±0.42	10.24±0.78^*^	27.58±0.87^#^	6.22±0.32^*^
LVSP (mmHg)^a^	170.5±3.7	123.5±5.3^*^	98.8±3.1^#^	150.5±3.6^*^
LVdp/dtmax(mmHg/s)^a^	2959±69	2136±70^*^	1113±48^#^	1749±49^*^
NT-proBNP (pg/ml)^b^	50(21~289)	150(28~589) ^*^	220(108~1589) ^#^	450(128~2589)^*^

Data are presented as mean± standard deviation ^a^ and/or median (range)^b^. ^*^ P<0.05 versus ND-Sham, ^#^ P<0.05 versus D-Sham. ND-Sham: Non-diabetes sham group; ND-IRI: Non-diabetes ischemia reperfusion injury; D-IRI: Diabetes ischemia reperfusion injury; D-Sham: Diabetes sham group.

**Figure 1:**
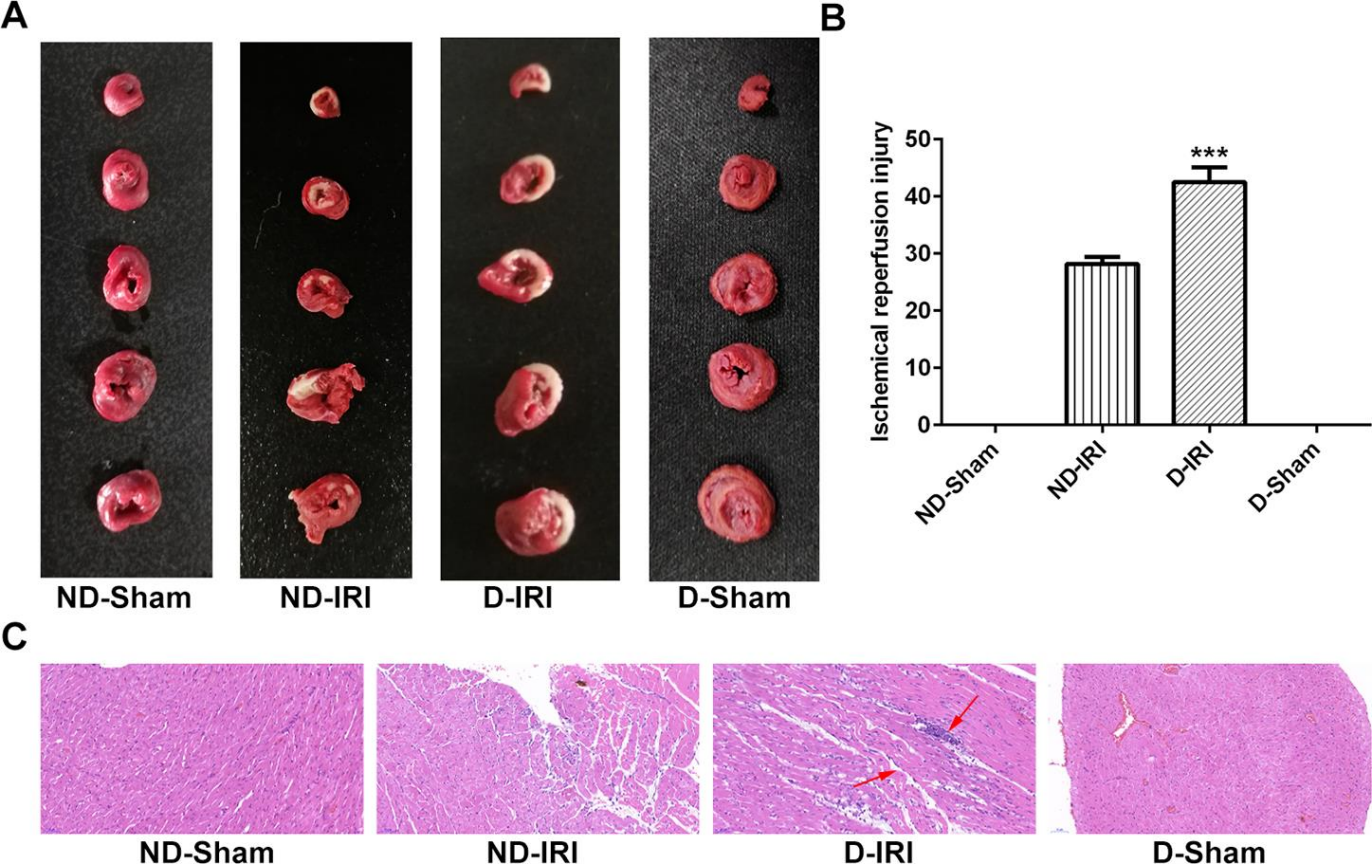
**Examination for the cardiomyocyte’s function in ischemia reperfusion (IR) injury.** (**A**, **B**) The IR injury was detected by the TTC staining. P<0.001 compared to ND-IRI group. (**C**) The cardiac pathology was evaluated by HE staining. Magnification, ×200. TTC: triphenyltetrazoliu chloride; HE: Hematoxylin-eosin; ND-Sham: Non-diabetes sham group; ND-IRI: Non-diabetes ischemia reperfusion injury; D-IRI: Diabetes ischemia reperfusion injury; D-Sham: Diabetes sham group.

As shown in Figure 1A, 1B, TTC staining showed that the infarct size in D-IRI group was significantly increased compared to the ND-IRI group. Moreover, there was no obvious infarct field in ND-Sham and D-Sham groups. In the IR group, loss of myofibril, cardiomyocytes necrosis and abnormal structure emerged (Figure 1C). These results indicated that the cardiac function dramatically descended and the model was established successfully.

### D-IRI injury associated with apoptosis

**Figure 2:**
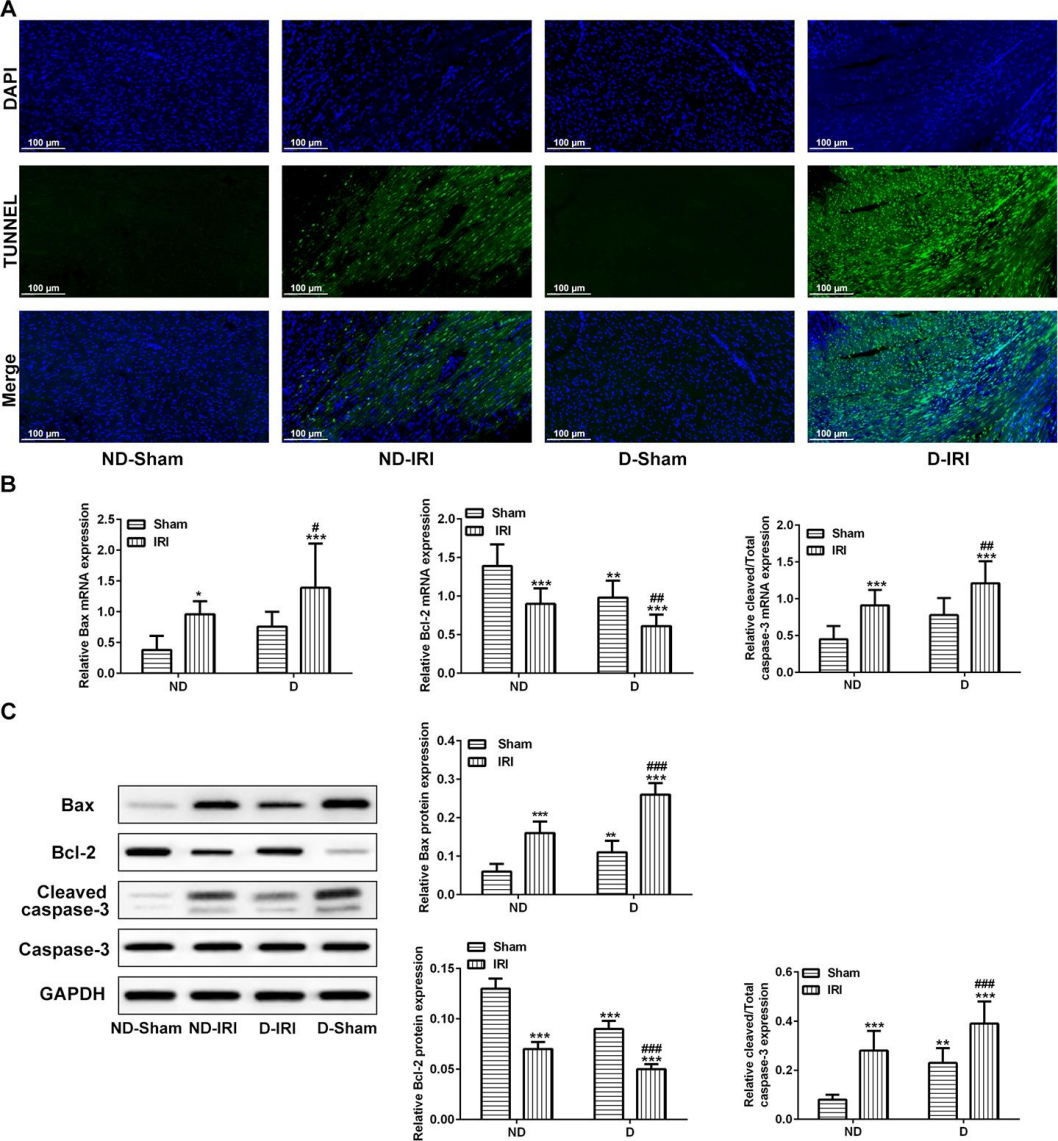
**Observation for the apoptosis and associated biomarkers.** (**A**) Apoptosis in myocardium was determined by TUNEL analysis. Scale bar=50 μm. (**B**) mRNA levels of Bcl-2, Bax, and cleaved caspase-3 detecting by RT-qPCR. ^*^P<0.05, ^**^P<0.01, ^***^P<0.001 represents the values compared to the ND-Sham group; ^#^P<0.05, ^##^P<0.01 represents the values compared to D-Sham. (**C**) Protein levels of Bcl-2, Bax, and cleaved caspase-3 detecting by western blot assay. ^**^P<0.01, ^***^P<0.001 represents the values compared to the ND-Sham group; ^###^P<0.001 represents the values compared to D-Sham. ND-Sham: Non-diabetes sham group; ND-IRI: Non-diabetes ischemia reperfusion injury; D-IRI: Diabetes ischemia reperfusion injury; D-Sham: Diabetes sham group.

As shown in Figure 2A, apoptosis detected by TUNEL assay was observed in ND-IRI and D-IRI groups but not in ND-Sham and D-Sham groups, and D-IRI rats presented more significant increase of apoptosis level than ND-IRI rats. According to the above results, we speculated that the key biomarkers of apoptosis might participate in the pathological processes of D-IRI or ND-IRI. The expression of Bax, Bcl-2 and cleaved caspase-3 (activated form of caspase-3) was examined by RT-qPCR and western blot assays. The RT-qPCR results revealed that Bcl-2 was significantly reduced in ND-IRI and D-IRI groups compared to ND-Sham and D-Sham groups, respectively (Figure 2B). Meanwhile, the Bcl-2 expression in D-IRI group was remarkably decreased compared to ND-IRI group (Figure 2B, P<0.001). However, Bax and cleaved caspase-3 expression were remarkably increased in ND-IRI and D-IRI groups compared to ND-Sham and D-Sham groups, respectively. The expression changes in D-IRI group were more than those in ND-IRI group (Figure 2C). Moreover, the western blot assay also illustrated the same changes of Bcl-2, Bax and cleaved caspase-3 (Figure 2C). These results indicated that apoptosis might be involved in D-IRI.

### Alteration of Apelin levels in myocardial tissues and serum in D-IRI and ND-IRI rats

**Figure 3:**
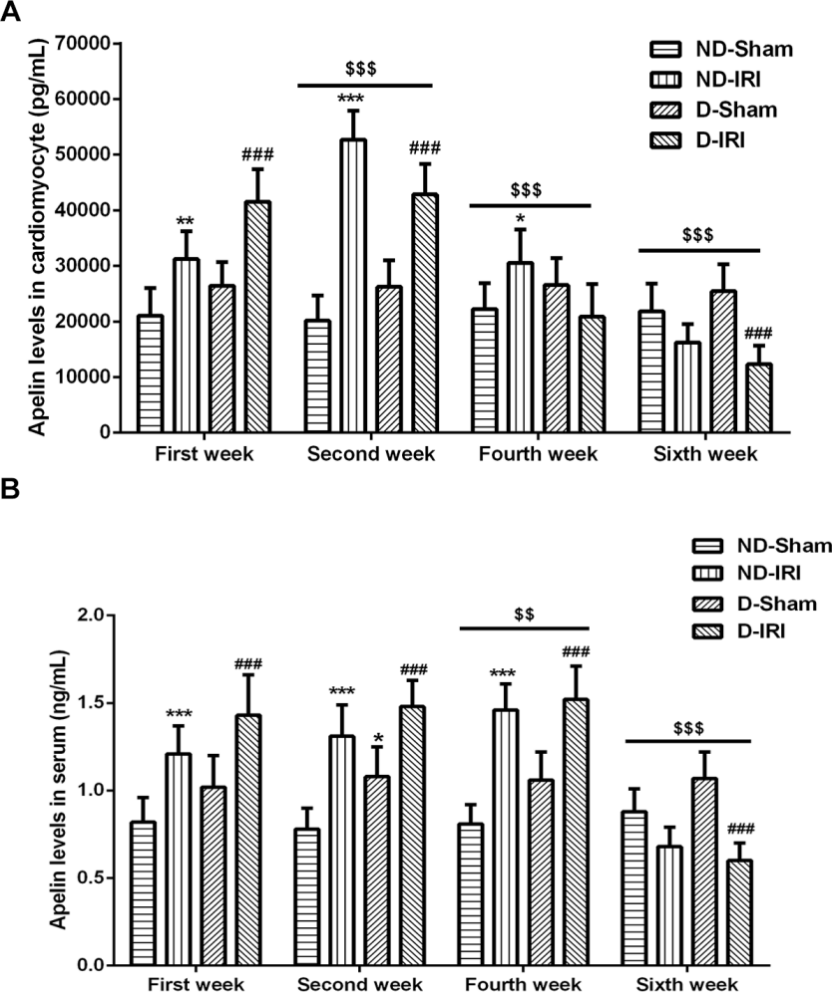
**Evaluation for the levels of Apelin in diabetic- or no diabetic IRI rats.** The levels of Apelin in both cardiomyocyte (**A**) and serum (**B**) were evaluated by ELISA kit. ^*^ P<0.05, ^**^P<0.01, ^***^P<0.001 represent the values compared to the ND-Sham group. ^###^ P<0.001 represents the values compared to the D-Sham group. ^$$$^P<0.001 represents the values compared to the First week. ND-Sham: Non-diabetes sham group; ND-IRI: Non-diabetes ischemia reperfusion injury; D-IRI: Diabetes ischemia reperfusion injury; D-Sham: Diabetes sham group.

In order to discover a biomarker that triggers the diabetic myocardial ischemia reperfusion injury, the cardiovascular disease-related protein, Apelin, was examined in both myocardial tissues and serum. In the former two weeks, the expression levels of Apelin in myocardium tissues were increased in ND-IRI and D-IRI groups compared with ND-Sham and D-Sham groups (Figure 3A). Also, the Apelin levels were expressed more in D-IRI group than in ND-IRI group (Figure 3A). However, the Apelin expression in ND-IRI, D-Sham and D-IRI was significantly lowered from the fourth week (Figure 3A). Meanwhile, the blood samples were used to detect expression level of Apelin in serum, which illustrated the similar changes to myocardial tissues. However, the decrease of Apelin in serum was delayed for two weeks compared to myocardial tissues (Figure 3B).

### Apelin improved cardiac function for D-IRI rats

The ultrasonic inspection was performed 6 weeks after surgery. The results unveiled that the LVIDd, LVIDs and LVPWs were significantly higher, whereas the EF and FS were significantly lower in the D-IRI control group and Ad-GFP group compared to the D-Sham group (Table 3). Further, the LVIDd, LVIDs, and LVPWs were significantly lower, and the EF and FS were significantly higher in Ad-Apelin compared to Ad-GFP group (Table 3).

**Table 3:** Ultrasonic inspection results for the diabetes ischemia reperfusion injury rat models after Apelin therapy.

**Group**	**D-Sham (n=7)**	**D-IRI (n=14)**	**Ad-GFP (n=6)**	**Ad-Apelin (n=6)**
LVIDd (mm)	9.35±0.53	15.321±0.82^*^	14.88±0.86^*^	9.23±0.56^#^
LVIDs (mm)	4.02±0.68	8.89±0.98^*^	9.63±0.89^*^	4.22±0.68^#^
EF (%)	56.58±3.22	35.73±2.88^*^	35.43±3.96^*^	55.65±3.22^#^
FS (%)	40.13±4.35	26.82±4.39^*^	27.44±3.85^*^	39.31±3.87^#^
IVSd (mm)	1.44±0.45	1.83±0.59	1.98±0.55	1.49±0.48
IVSs (mm)	2.06±0.55	3.02±0.63	3.11.11±0.51	1.96±0.46
LVPWd (mm)	1.92±0.26	2.15±0.17	2.35±0.25	1.98±0.26
LVPWs (mm)	2.47±0.18	3.07±0.22^*^	3.01±0.20^*^	2.87±0.15^#^

^*^ P<0.05 represents the values compared to the D-Sham group. ^#^ P<0.05 represents the values compared to Ad-GFP. D-Sham: Diabetes sham group. D-IRI: Diabetes ischemia reperfusion injury; Ad-GFP: D-IRI rats injecting Ad-recombined lentiviral vector expressing GFP. Ad-Apelin: D-IRI rats injecting Ad-recombined lentiviral rector expressing Apelin.

**Figure 4:**
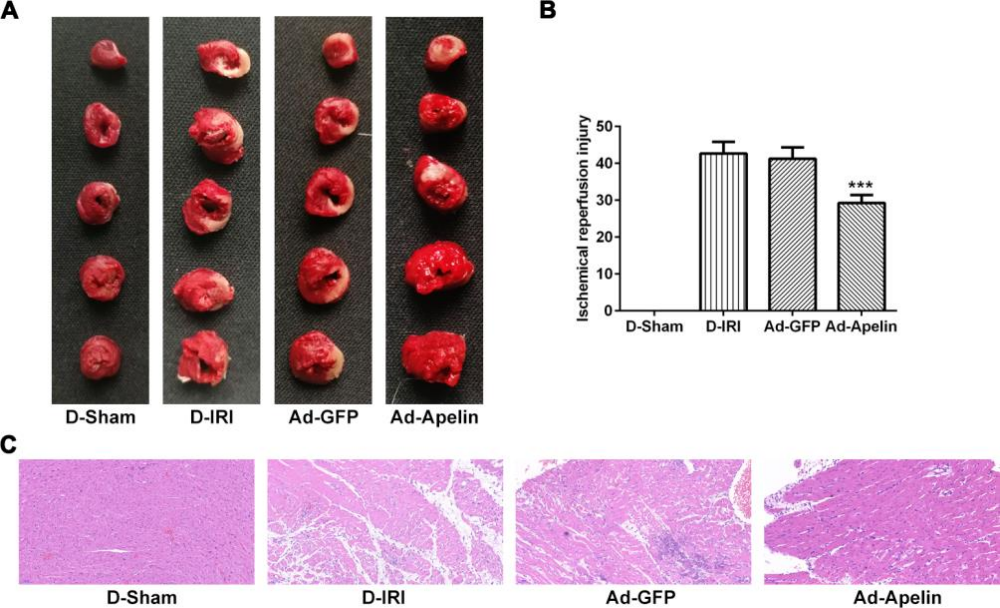
**Role of Apelin on cardiac infarction and pathology in cardiac IRI rats.** (**A**, **B**). The infarction of cardiac was evaluated by TTC staining. ^***^P<0.001 versus D-IRI. (**C**) The pathology of myocardium was assessed by HE staining. Magnification, ×200. TTC: triphenyltetrazoliu chloride; HE: Hematoxylin-eosin; D-Sham: Diabetes sham group; D-IRI: Diabetes ischemia reperfusion injury; Ad-GFP: D-IRI rats injected with Ad-recombined lentiviral vector expressing GFP. Ad-Apelin: D-IRI rats injected with Ad-recombined lentiviral rector expressing Apelin.

After the injection of the Ad-Apelin, the hemodynamic parameters, including LVEDP, LVSP and LVdp/dtmax, were assessed. IRI injury significantly increased the LVEDP and decreased LVSP and LVdp/dtmax levels in D-IRI and Ad-GFP groups compared to D-Sham group (Table 4). The results indicated that the LVEDP was decreased notably in Ad-Apelin group compared to Ad-GFP group (Table 4). Meanwhile, the LVSP and LVdp/dtmax were increased notably in Ad-Apelin group in comparison with the D-IRI control group and Ad-GFP group (Table 4). Test of NT-proBNP unmasked that the NT-proBNP levels were decreased markedly compared to D-IRI control group and Ad-GFP group (Table 4). Furthermore, the indexes for IRI, including IRI size and heart weight index, were also evaluated. The results discovered that the IRI size in myocardial tissues of Ad-Apelin group was significantly less than those of D-IRI control and Ad-GFP groups (Figure 4A, 4B). Meanwhile, the heart weight index was also decreased in Ad-Apelin group compared to Ad-GFP group (Table 4). In the IR group, Apelin administration reversed the D-IRI injury on the loss of myofibril, cardiomyocytes necrosis and abnormal structure (Figure 4C).

**Table 4:** Cardiac hemodynamic for the diabetes ischemia reperfusion injury rat models after Apelin therapy.

**Group**	**D-Sham (n=7)**	**D-IRI (n=14)**	**Ad-GFP (n=6)**	**Ad-Apelin (n=6)**
Body weight (g) ^a^	248.1±4.1	255.1±3.8	250.2±3.8	255.6±3.9
Heart weight (g) ^a^	1.041±0.049	1.682±0.051	1.692±0.041	1.208 ±0.045
HWI ^a^	0.0041±0.0001	0.0066±0.0004^*^	0.0067±0.0004^*^	0.0047±0.0002^#^
LVEDP (mmHg)^a^	6.22±0.32	27.58±0.87^*^	28.44±0.98^*^	9.06±0.57^#^
LVSP (mmHg)^a^	150.5±3.6	98.8±3.1^*^	99.5±4.3^*^	146.7±4.66^#^
LVdp/dtmax(mmHg/s)^a^	1749±49	1113±48^*^	1256±40^*^	1819±64^#^
NT-proBNP^b^	450(128~2589)	220(108~1589)^*^	240(115~1789)^*^	450(128~2589)^#^

Data are presented as mean ± standard deviation ^a^ and/or median (range)^b^. ^*^P<0.05 versus D-Sham, ^#^P<0.05 versus Ad-GFP. HWI, heart weight index. D-Sham: Diabetes sham group. D-IRI: Diabetes ischemia reperfusion injury; Ad-GFP: D-IRI rats injecting Ad-recombined lentiviral vector expressing GFP. Ad-Apelin: D-IRI rats injecting Ad-recombined lentiviral rector expressing Apelin.

### Apelin enhanced PPAR levels and decreased collagen volume fraction (CVF) value in myocardial tissues

**Figure 5:**
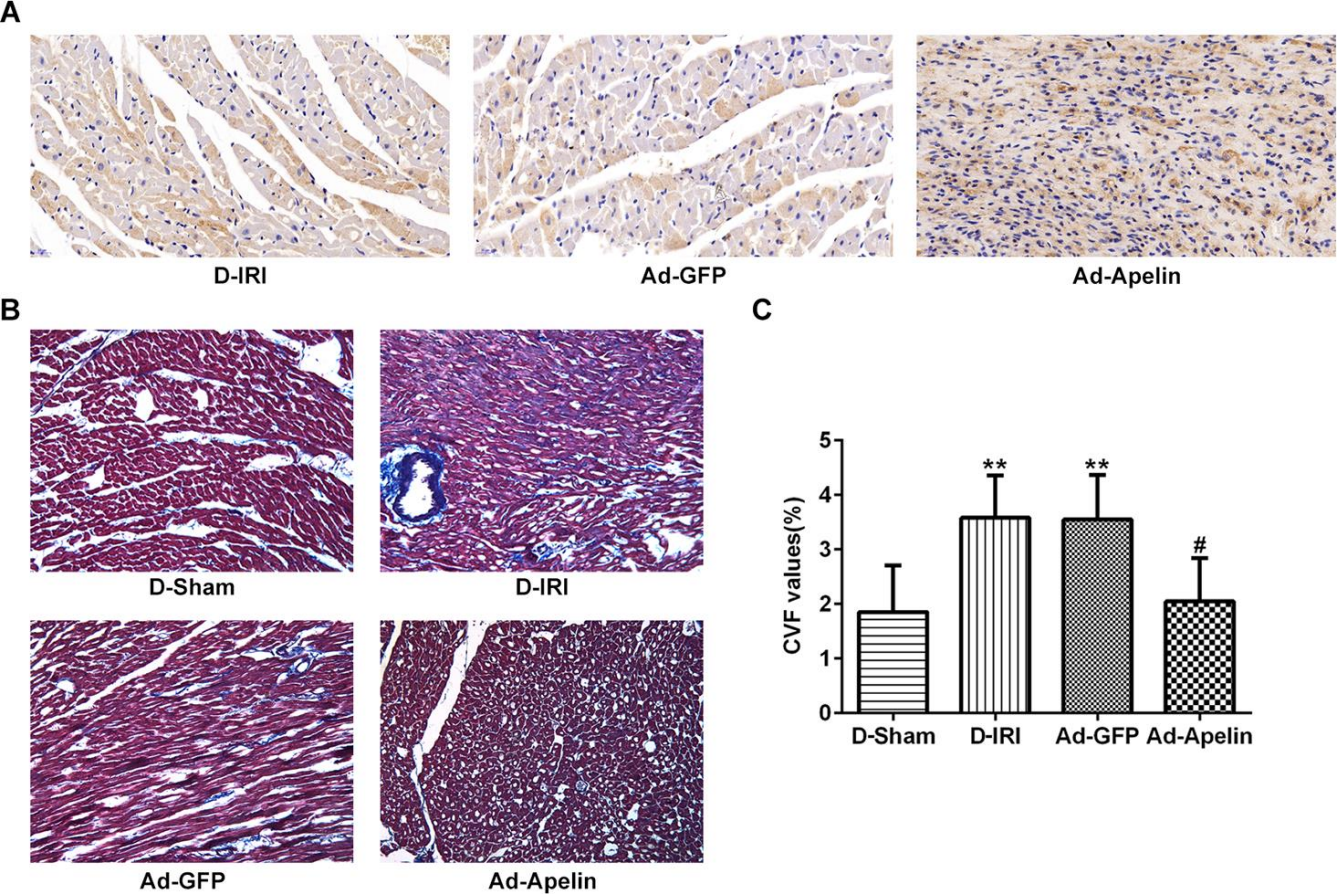
**Impacts of Apelin on PPAR-alpha expression and CVF values in D-IRI rats.** (**A**) Examination for PPAR-alpha expression by immunohistochemistry staining. Magnification, ×200. (**B**, **C**) CVF values in D-IRI rats was examined by Masson’s staining. ^**^ P<0.01 represents the values compared to the D-Sham group. ^#^P<0.05 represents the values compared to the Ad-GFP group. Magnification, ×200. CVF: collagen volume fraction; D-Sham: Diabetes sham group; D-IRI: Diabetes ischemia reperfusion injury; Ad-GFP: D-IRI rats injected with Ad-recombined lentiviral vector expressing GFP. Ad-Apelin: D-IRI rats injected with Ad-recombined lentiviral rector expressing Apelin.

To investigate the protective role of Apelin in cardiovascular diseases, the PPAR-alpha and CVF were examined in the myocardial tissues. The results exhibited that the PPAR-alpha expression was dramatically elevated in the Ad-Apelin group relative to the Ad-GFP group (Figure 5A). The Masson’s stain evaluated the myocardial fibrosis level, and CVF value was significantly lessened in the Ad-Apelin group in contrast to Ad-GFP group (Figure 5B, 5C).

### Apelin inhibited apoptosis in D-IRI injury

**Figure 6:**
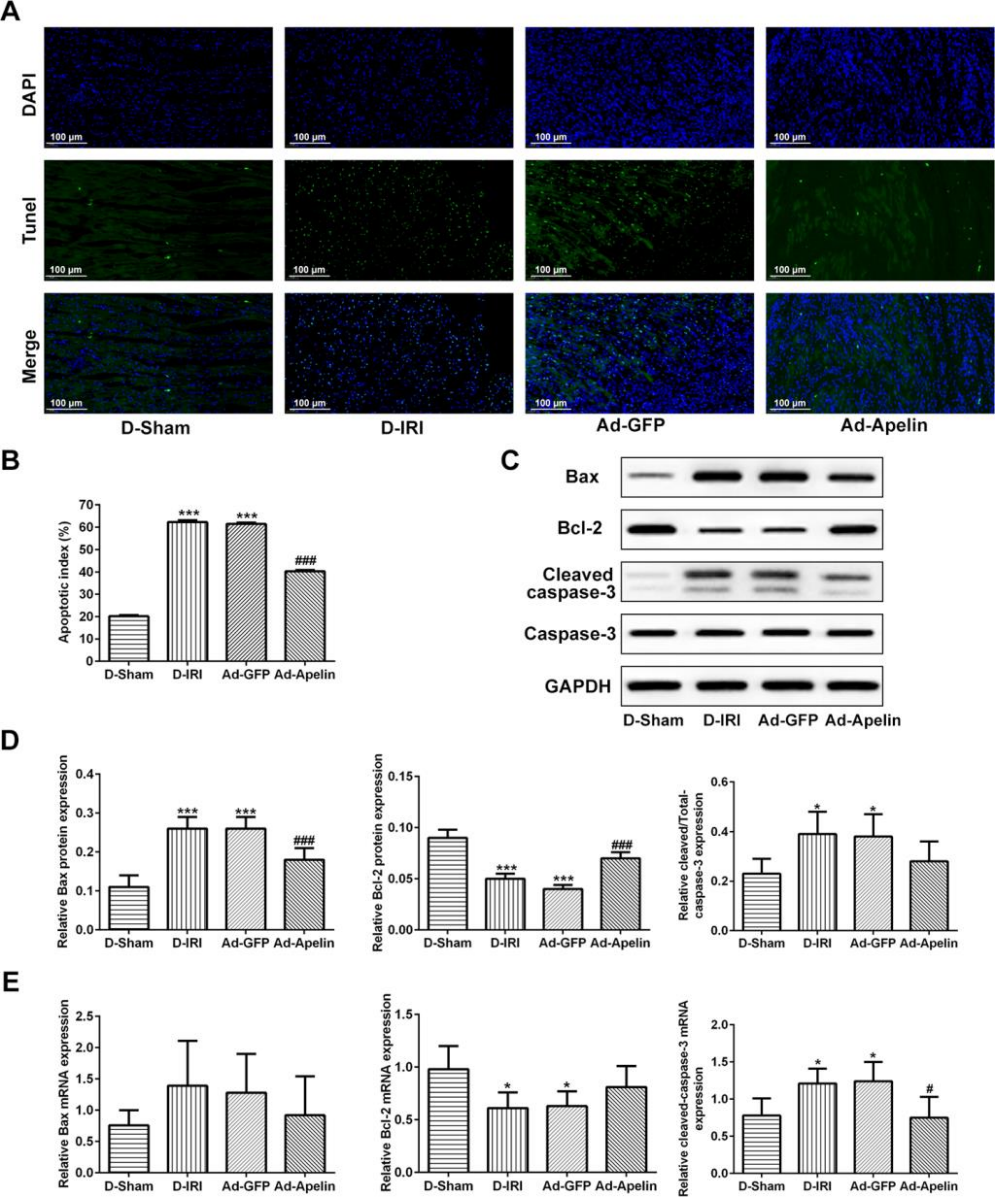
**Observation for the apoptosis reduced by the injection of recombined lentiviral vector expressing Apelin.** (**A**, **B**) Apoptosis in myocardium was determined by TUNEL analysis. Scale bar=50 μm. (**C**, **D**) Protein levels and analysis of Bcl-2, Bax, and cleaved caspase-3 detecting by western blot assay. (**E**) mRNA levels of Bcl-2, Bax, and cleaved caspase-3 detecting by RT-qPCR. ^*^ P<0.05, ^***^ P<0.001 represent the values compared to the D-Sham group. ^###^P<0.001 represents the values compared to Ad-GFP group. D-Sham: Diabetes sham group; D-IRI: Diabetes ischemia reperfusion injury; Ad-GFP: D-IRI rats injected with Ad-recombined lentiviral vector expressing GFP. Ad-Apelin: D-IRI rats injected with Ad-recombined lentiviral rector expressing Apelin.

In order to investigate the mechanism of the protective function of Apelin on the myocardial tissues, the apoptosis level and apoptotic biomarkers, such as Bax, Bcl-2 and cleaved caspase-3, were examined by the TUNEL, RT-qPCR and western blot assays, respectively. The TUNEL staining results indicated the reduced apoptotic index in Ad-Apelin group compared to Ad-GFP group (Figure 6A, 6B). The western blot analysis results indicated that the levels of Bax and cleaved caspase-3 were significantly declined, and Bcl-2 levels were significantly elevated compared to the Ad-GFP group (Figure 6C, 6D). Likewise, in RT-qPCR analysis, the decrease of Bcl-2 and increase of cleaved caspase-3 in D-IRI group and Ad-GFP group compared to D-Sham group were viewed, whereas the cleaved caspase-3 expression was inhibited after administration of Ad-Apelin (Figure 6E). Although the gene alteration of Bax and Bcl-2 was not significant in some groups, the increase or decrease trend was similar to protein changes.

### Apelin played roles in a few signaling pathways

**Figure 7:**
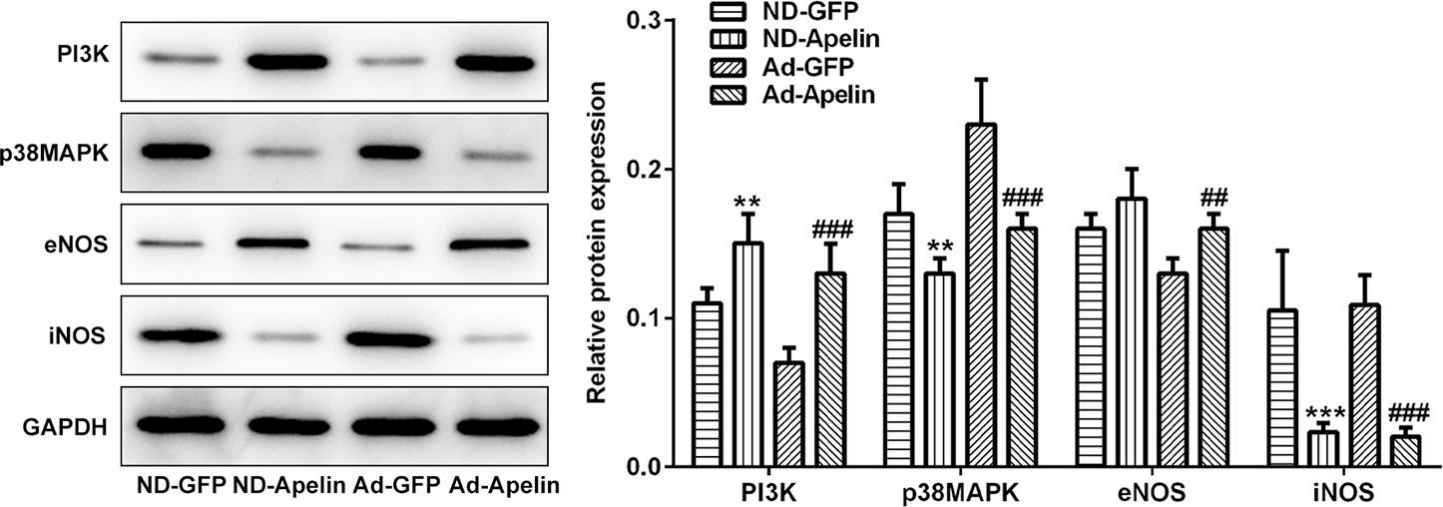
**Examination for expression of PI3K, eNOS, iNOS and p38MAPK.** (**A**) The blotting bands and statistical analysis for PI3K, p38MAPK, eNOS and iNOS expression examined by western blot assay. ^**^P<0.01, ^***^P<0.001 represent the values compared to the ND-GFP group. ^##^P<0.01, ^###^P<0.001 represent the values compared to the Ad-GFP group. ND-GFP: Non-diabetes ischemia reperfusion injury rats injected with recombined lentiviral vector expressing GFP; ND-Apelin: Non-diabetes ischemia reperfusion injury rats injected with Ad-recombined lentiviral rector expressing Apelin; Ad-GFP: Diabetes ischemia reperfusion injury rats injected with Ad-recombined lentiviral vector expressing GFP. Ad-Apelin: Diabetes ischemia reperfusion injury rats injected with Ad-recombined lentiviral rector expressing Apelin.

To clarify the specific pathway by which the Apelin protected against the IRI, the PI3K, p38MAPK, eNOS and iNOS expression levels were examined using the western blot assay (Figure 7). The PI3K levels were significantly enhanced in Apelin group compared to the GFP group. Furthermore, the p38MAPK and iNOS expression was repressed in Apelin group compared to GFP group.

## DISCUSSION

The diabetes mellitus-caused cardiovascular diseases are the most important reasons for the higher mortality of humans, especially the type 2 diabetes mellitus [24]. Among these diseases, the morbidity of D-IRI is increasing with each passing year [25, 26]. The previous study [27] indicated that the diabetes mellitus can cause many dysfunction, endothelial dysfunction, insulin resistance, abnormal metabolism of myocardium included. In the recent years, it has been reported that the initial processes for the diabetic myocardial remodeling and the myocardial diastolic dysfunction generate cardiac insufficiency. Schannwell et al. [28] performed a large epidemiological investigation and discovered that the morbidity and mortality of cardiovascular diseases in diabetes mellitus patients are significantly higher compared with non-diabetic patients. Therefore, we established the diabetes mellitus model and/or IRI model to compare the alterations of cardiac functions and apoptotic characteristics.

The ultrasonic inspection results showed that the LVIDd, VLIDs, LVPWs and LVEDP were increased significantly, but LVSP and LVdp/dtmax were significantly decreased in ND-IRI (or D-IRI) group compared to ND-Sham (or D-Sham) group. The result hinted that the chamber of heart was significantly enlarged, and that left ventricular systolic thickness significantly decreased. Meanwhile, the EF and FS were reduced markedly and LVEDP was increased significantly in IRI rats, suggesting that heart function decreased significantly. Meanwhile, LVIDd, VLIDs, and LVPWs were augmented, whereas the EF and FS were lessened significantly in D-IRI group compared to ND-IRI group. These results suggested that the heart failure was aggravated in the D-IRI rats compared to ND-IRI rats. Therefore, we believed that the symptoms of heart failure were more serious in D-IRI rats than ND-IRI rats.

Past studies [29, 30] have proved that the NT-proBNP is the optimized biomarker for the diagnosis of heart failure, and is the optimized biomarker for prognosis of cardiovascular diseases. The results indicated that NT-proBNP concentration in D-IRI group was markedly increased compared to D-Sham group but increased significantly compared to ND-IRI group. These findings hinted that the rats had developed the decreased cardiac functions before the formation of the cardiac infarcts in D-IRI rats. We speculated that the D-IRI caused high hypoxia, leading to the enlarged ventricle and increased secretion of NT-proBNP, consistent with the previous study [31].

Recently, researches [32, 33] have found that the apoptosis participates in processes of the physiological and pathological changes following with the development of study of cardiovascular diseases. Herein, we examined the key biomarkers [34], such Bax, Bcl-2 and cleaved caspase-3 (activated form of caspase-3), by using RT-qPCR and western blot experiments. The changes of Bax and Bcl-2 illustrated that the hypoxia-ischemia induced in D-IRI rats and ND-IRI rats activated apoptosis. The mechanism of the apoptosis may be triggered by the oxidative stress and decreased mitochondrial potential, which sequentially induces the release of cytochrome C [35]. Subsequently, the cytochrome C provokes the cleavage of the caspase-3 to the activated form, cleaved caspase-3.

Apelin and its receptor, AJP, are extensively distributed in heart and the vascular tissues, both of which involve in various pathological processes, especially in the cardiovascular disorders, diabetes complicated with micro-vascular diseases and the ischemia reperfusion injuries [20]. Therefore, in this study, the levels of Apelin were raised significantly in the early stage of the IRI in both D-IRI rats and ND-IRI rats, but decreased in the medium stage. However, the Apelin levels increased to the peak values at one week post the operation. According to the above results, we speculated that the diabetes and/or IRI-induced hypoxia-ischemia activated and the metabolic disturbance seriously upregulated the expression of the Apelin, and aggravated the cardiac function quickly. Our result was consistent with the previous study [36], showing that the Apelin played an important role in the early stage of heart failure. Thus, the expression of Apelin might be associated with the oxidative stress or apoptosis in the myocardial tissues. Considering all of the above results in both D-IRI and ND-IRI rats, including enlarged infarct size, enhanced apoptosis, decreased Apelin levels in D-IRI rats compared to ND-IRI rats, we concluded that the cardiac function in D-IRI rats was more serious compared to the ND-IRI rats. Therefore, in the following study, we mainly investigated the effects of exogenously expressed Apelin on the cardiac function and the associated indexes.

According to the observation of echocardiographic measurements and cardiac hemodynamic measurements, we discovered that the exogenously expressed Apelin significantly improved the cardiac functions. Meanwhile, the ischemia reperfusion injury size and heart weight index were significantly decreased under Apelin treatment. The above results suggested that the enhanced levels of Apelin in cardiac tissues might be a kind of compensatory mechanism for the cardiovascular diseases [37].

Actually, multiple mechanisms of the diabetic myocardial ischemia reperfusion injury have been clarified, and many biomarkers for the prognosis of IRI in diabetes patients have also been discovered [38–40]. The peroxisome-proliferator-activated receptors (PPARs) are gene transcriptional regulators, which could be activated and modulate the transcription of many genes associated with insulin effects. The PPARs mainly regulate the lipid metabolism, anti-oxidant defense, mitochondrial biogenesis, endothelial cell metabolism, and also play protective functions in cardiovascular diseases, atherosclerosis, inflammatory diseases, among others [41, 42]. Chen et al. [43] found that the PPAR functions in many biological processes of the cardiovascular system, and antagonizes the hypertension and heart failure. In this work, the PPARα levels were significantly enhanced under treatment with the Apelin, suggesting that the PPAR plays a critical role in the rescue of the cardiac function in D-IRI rats and may be an important mechanism of anti-heart failure in D-IRI rats.

In order to investigate the effects of Apelin on the apoptosis of cardiac tissues, the Bax, Bcl-2 and cleaved caspase-3, were detected in the Apelin overexpression rats and control rats. The results unmasked that Apelin addition significantly enhanced Bcl-2 levels and decreased Bax and cleaved caspase-3 levels, hinting that the Apelin could inactivate apoptosis. However, the apoptotic pathways always involve some of the biomarkers and molecules. The previous studies [44, 45] also elucidated that the oxidative stress together with some of its producers, such as reactive oxygen species (ROS), reactive nitrogen species (RNS), oxidases (NOXs), endothelial nitric oxide synthase [46], is associated with the major pathological progressions of the heart failure, ischemia-reperfusion injury and the cardiac hypertrophy. Except for the above biomarkers, several signaling transduction pathways, such as PI3K/Akt pathway [47], p38MAPK [48] and PI3K pathway [49], also participate in the cardiovascular diseases. In the present study, the overexpression of Apelin in D-IRI rats significantly enhanced the levels of PI3K and eNOS levels, and the production of the NO was also observed in D-IRI rats compared to the rats without Apelin treatment. We speculated that the Apelin enhanced the expression of PI3K and eNOS protein, thus triggering the production of NO. The increased NO levels could lead to the blood vessel diastolic and decrease the blood pressure. Moreover, the overexpression of Apelin also hindered the p38MAPK expression and decreased the apoptotic index in D-IRI rats.

Taken together, these data demonstrated that the Apelin overexpression protects against the diabetic myocardial ischemia reperfusion injury by inhibiting apoptosis and oxidative stress via PI3K and p38MAPK pathway in diabetic IRI rats. These findings provide critical new insight into the knowledge of Apelin's cardio-protective effects, and may develop a novel therapeutic strategy for the diabetic myocardial ischemia reperfusion injury patients. However, the mechanistic study, such as the PI3K inhibitor or P38 activator delivered in Apelin-treated D-IRI group, needs to be further strengthened in the following investigation, which is a limitation of the present study.

## MATERIALS AND METHODS

### Reagents and materials

Adult male Sprague-Dawley (SD) rats (body weight 200~270g) were purchased from Experimental Animal Center of Zhengzhou University, Zhengzhou, China. All rats were kept in individual cages under the controlled ambient temperature of 25 ± 2° C and humidity of 50 ± 5%. All rats were housed in a controlled pathogen free environment in a 12-h light-dark cycle. The rats were used to establish the animal models of DM, myocardial IRI and DM complicated with IRI, which would be applied in the following experiments. Male SD rats were randomly divided in two groups, the DM group and non-DM group. All experiments were performed according to the Guidelines of the Institutional Animal Care and approved by the Ethics committee of the Zhengzhou University, Zhengzhou, China.

### Type 2 diabetic mellitus (T2DM) rat model

The T2DM model was constructed by high-fat diet (HFD) and streptozotocin (STZ) injection, according to the previously published article [50], with a few modifications. Male SD rats were fed with HFD containing 62% basic fed, 15% cooked lard, 2% sugar, 8% whole milk powder, 8% hen yolk powder, 4% casein and 1% saline for 6 weeks. 30 mg/kg (Sigma-Aldrich, St. Louis, MO, USA) STZ (dissolved in the 0.1 mol/L citric acid, and adjusted to pH 4.2) was injected into the rats (fasting for 12 h) via intraperitoneal injection for one-time after 6 weeks of HFD feeding. The rats were given HFD continuously till the eighth week. Rats with fasting plasma glucose (FPG) levels after STZ injection more than 7.0 mmol/L and /or at 2 h time-point of PG more than 11.1 mmol/L were considered as T2DM model rats. There were 45 rats as the T2DM model rats in this study.

### Myocardial ischemia/reperfusion (IR) injury model

The normal rats and the T2DM rats were randomly and respectively divided into two groups, one is for IR injury model (D-IRI, n=14; ND-IRI, n=13) and another for Sham group (D-Sham, n=7; ND-Sham, n=8). The Apelin administration for IR model was divided into two groups, one is Ad-GFP control (n=6) and the other is Ad-Apelin (n=6). The IRI rats were anesthetized by using the ketamine hydrochloride (70 mg/kg) via intraperitoneal injection (i.p), and then the rats were fastened in a supine position. The rats were mechanically ventilated with air through a rodent ventilator followed by oral endotracheal intubation. The body temperature of the rats kept at 37 ± 0.5° C. The thoracic cavity was opened between the fourth and the fifth intercostal of the left edge of the thoracotomy, and the heart of the rat was exposed after pericardial incision. A 7-0 silk thread was sutured around the left anterior descending coronary artery of the rat, 2 mm from the tip of the left auricle. Ten minutes later, when the thread was clamped and tightened, a snare blocking the artery was formed. After ligation, regional epicardial cyanosis in the heart symbolized the successful ischemia. Sixty minutes after the ischemia, the coronary artery reperfusion was performed by releasing the clamp for 1 h. Meanwhile, the Sham-operated animals were performed with the same surgical procedures, although the suture was not fastened. The rat chests were closed post the reperfusion step by step [51]. When the spontaneous breathing of rats was recovered, the breathing machine was removed. These rats were fed at the temperature of 25 ± 2° C and humidity of 50 ± 5%.

### Ultrasound measurements

After intervention, echocardiographic analysis was performed to detect the hypertrophic degree of heart with rats in a supine position. Rats were anesthetized by injecting 10% chloral hydrate. Then, the rats were placed on the isothermal pad at the temperature of 40° C. The liner array ultrasound transducer probe (9.0 Hz) was employed to examine the echocardiographic recordings. The long axis views from the left ventricle (LV) were obtained to measure the left ventricular internal diastolic diameter (LVIDd), left ventricular internal dimension systole (LVIDs); the short axis views were used to examine left ventricular posterior wall depth at end-diastole (LVPWd) and left ventricular posterior wall depth at end-systole (LVPWs); left ventricular septal depth at end-diastolic (LVSd), left ventricular septal depth at end-systole (LVSs); ejection fraction (EF, %) and fractional shortening (FS, %) were calculated according to CUBED formula, as previously described [52]. All measurements were repeated three times from at least 10 consecutive cardiac cycles, as the recommendations of American Society of Echocardiography described [53].

### Cardiac hemodynamic evaluations

During surgery, the LabScribe software was applied to record and analyze hemodynamic parameters including LV peak systolic pressure (LVSP), the largest upstroke or descendent velocity of the LV pressure (±dp/dt max) and LV end-diastolic pressure (LVEDP). The LV hemodynamics data was obtained by using the pressure volume catheter following the previously published study [54]. In brief, the right-side carotid artery was cannulated by using the pressure volume catheter after anesthesia (i.p. with the ethyl carbamate, 0.75 g/kg, and α2 chloralose, 70 mg/kg). Then, the tip of the pressure volume catheter was inserted into the left side ventricle via the aorta.

### Infarct size measurement

The heart was rapidly excised and frozen at -20° C after the cardiac hemodynamic measurements. Then the frozen heart was cut into 2 mm thick sections. Slices were incubated in the 0.1% triphenyltetrazoliu chloride (TTC) for 10 min at 37° C in phosphate buffer (pH 7.4). The stained slices were subsequently fixed by using the 10% formaldehyde for one night and scanned according to the previous paper [55]. TTC-unstained pale area (infarct area, IA) and TTC-stained red area (ischemic but viable myocardium, IV) were evaluated through planimetry with the image analysis software. Myocardial infarct size was detected as the percent of infarct area over total myocardium area (IV% = IV/(IA+IV) ×100%).

### NT-proBNP examination

The blood samples for examination of the NT-proBNP were collected by venipuncture. The blood samples were centrifugated at the speed of 3000 r/min for 10 min at 4° C to obtain the serum, which was shock-frozen and stored at the temperature of -80° C. Then NT-proBNP levels were measured utilizing the electrochemiluminescence immunoassay (ECLIA) and NT-proBNP detection kit (Roche, USA), and detected with the Roche Elec sys 2010 (Roche, USA). The lower to upper limit of the rats were 5 to 35000 pg/ml.

### Terminal deoxynucleotidyl transferase-mediated dUTP nick end labeling (TUNEL) staining

The TUNEL staining could examine the 3’-OH terminals of damaged DNA in late apoptosis cells. Therefore, the TUNEL was used to observe apoptosis level in the myocardial infarct size of rats. The TUNEL assay was performed by utilizing the TUNEL-POD kit (Roche, Germany), as previously described [56]. Briefly, the isolated myocardial tissues were embedded with paraffin and sectioned into slices soaked and washed by xylene and hydrated in gradient alcohol. Then the paraffin section of myocardial tissues was treated by proteinase K working solution for 30 min, with PBS washing for three times (3 min/wash). The 3% H_2_O_2_ solution was used to inactivate the endogenous peroxidase in the myocardial tissues for 10 min, with three times (3 min/wash) of PBS rinse. The paraffin section was treated with TUNEL reaction mixture (biotin dUTP and terminal deoxynucleotidyl transferase (TdT)) in a dark box at 37° C for 1 h, washed by PBS three times. An equal volume of solution with biotin dUTP to replace the TUNEL reaction mixture was used as the negative control. Samples were analyzed under fluorescence microscope (Olympus, Japan) using an excitation wavelength in the range of 450 nm and detection in the range of 565 nm.

### Recombined lentiviral vector of Apelin and therapeutic trial

The high titer recombined lentiviral vector was purchased from Ben Yuan Zheng Yang Bio. Co. Inc. (Beijing, China). Meanwhile, the recombined lentiviral vector carrying Apelin and control-GFP were established by Ben Yuan Zheng Yang Bio. Co. Inc. (Beijing, China). D-IRI rats, which was divided into three groups, including D-IRI control group (normal saline (500 μl) via tail vein injection), Ad-GFP group (Ad-GFP vector (500 μl) via tail vein injection) and Ad-Apelin group (Ad-Apelin vector (500 μl) via tail vein injection). The tail vein injection of lentiviral vector carrying Apelin and control-GFP were given 24 h and 14 days after the IR surgery.

### Reverse transcription-quantitative polymerase chain reaction (RT-qPCR) analysis

The total RNA was extracted from the myocardium by utilizing TRIzol reagent (Invitrogen, CA, USA). In brief, the myocardium was lysed with TRIzol reagent (1 ml). Then, the isolated RNA was extracted with 200 μl chloroform (Invitrogen, CA, USA), and supernatants were stored for following experiments. The isopropanol (1 ml) was used to precipitate the RNA, following with washing in 70% ethanol for two times. Then, the RNA precipitations were dissolved in diethy pyrocarbonate (DEPC) pre-treated water. Consequently, the promising cDNAs were synthesized by utilizing the Reverse Transcription System (Promega, USA) and AMV reverse transcription enzyme (Promega, USA), RNase inhibitor and ddH_2_O, 1 μg of total tissue RNA. The reverse transcription condition was listed as below: 85° C for 35 min, 4° C for 10 min.

The RT-qPCR and analysis for the Bax, Bcl-2, cleaved caspase-3 and GAPDH amplification were performed according to previous researches [57, 58]. The primers for Bax, Bcl-2, cleaved caspase-3 and GAPDH were listed in Table 5 and cDNA was used as the template. The forward and reverse primers, 2×SYBR Green Mixture (Invitrogen, USA), cDNA and ddH2O were used for the RT-qPCR amplification. The conditions of PCR reaction included pre-denaturation at 94° C for 2 min, denaturation at 94° C for 20 s, annealing at 55-58° C for 40 s, extension at 72° C for 40 s for 32 cycles and extension at 72° C for 2 min. The RT-qPCR assay was conducted on the PCT-100 PCR cycler (MJ Co. Ltd., USA). Each sample was run in triplicate.

**Table 5:** The gene sequences for the RT-qPCR amplification.

**Genes**		**Sequences**
Bax	Forwards	5'-TTTGTTACAGGGTTTCATCCAGG-3'
	Reverse	5'-GCTAGTGTCTGCCATGTA TT-3'
Bcl-2	Forwards	5'-GGGATACTGGAGATGAAGACT-3'
	Reverse	5'-CCCACCGAAC TCAAAGAAGG-3'
cleaved	Forwards	5'-GCTGAACTGEGGTATTGAG-3'
caspase-3	Reverse	5'-CCTGGAACATCGGATTTGATT-3'
GAPDH	Forwards	5’-CCATCACCATCTTC C AGGAG-3'
	Reverse	5'- CCTGCTTCACCTTCTTG-3'

### Western blot analysis

The expression of Bax, Bcl-2, cleaved caspase-3, PI3K, p38MAPK, eNOS and iNOS was analyzed by western blot analysis, and samples was normalized to GAPDH, as previously described [59–61]. The myocardium were lysed utilizing the RIPA buffer (1 ml/100 mg) at 0° C, and centrifugated at the speed of 12000 r/min for 10 min. The supernatant (40 μg) was loaded to western blot assay by using 15% SDS-PAGE, and was electronic-transferred onto the polyvinylidene fluoride (PVDF) membrane (Dupont, USA). Then, the membranes were blocked utilizing 5% non-fat milk for 2 h at room temperature, followed by wash with PBST buffer for 3 times (5 min/wash). The membranes were incubated with primary antibodies at 4° C for whole night. The membranes were washed with PBST buffer for three times (5 min /time). Then, the membranes were cultivated with AP-labelled goat anti-rabbit IgG and AP-labeled goat anti-mouse IgG (1:10000, Santa Cruz, Co. Ltd. CA, USA) at 37° C for 60 min, and rinsed with PBST buffer for three times (5 min /time). Finally, the membranes were incubated with ECL reagent (Pierce, USA) in the dark for 3 min. The density of bands was evaluated by Image Analysis Software (Bio-Rad Co. Ltd., USA).

### ELISA assay for Apelin analysis

The blood samples were collected from the aorta abdominalis, and then formed the mixture with the Trasylol (0.6 TIU/ml). Then the blood samples were centrifugated at 1600 r/min for 15 min at 4° C, and the supernatant was prepared for the ELISA analysis. The serum levels of Apelin was examined by using the commercial Apelin-12 ELISA assay kit (Phoenix pharmaceutical Inc. USA) according to the instruments of the manufacturer.

### Immunohistochemistry assay

The immunohistochemistry was employed to assess PPAR-alpha and Apelin expression in the myocardium. The myocardial tissues were fixed utilizing 4% paraformaldehyde continuously for 24 h. Then, the tissues were embedded in paraffin, and sectioned into slices at 4 μm thick by using a rotary microtome. For the immunohistochemistry assay, the endogenous peroxidase was inactivated by incubation with 3% hydrogen peroxide, and slices were blocked by using the 5% BSA for 20 min. Then, the slices were incubated with the rabbit anti-rat PPAR-alpha polyclonal antibody (1:2000 in PBS) (Abcam, UK) at 4° C overnight. Subsequently, the slices were grown with goat anti-rabbit peroxidase-conjugated IgG (1:500 in PBS) (Abcam, UK) at 25° C for 1 h. Briefly, the immunohistochemistry was conducted with the commercial SP immunohistochemistry kit (Zymed, USA). Finally, the slices were immersed in alkaline phosphatase labeled diaminobenzidine (DAB, ZSGB Bio. Inc. Co., Beijing, China), and the images were captured by the inverted fluorescence microscope (Olympus, Japan). Finally, the images were analyzed by utilizing Medical Image Analysis System (HMIAS22000).

### Evaluation of collagen volume fraction

The isolation, fixation and the staining for the myocardial biopsy tissues were performed according to the previous study [62]. Among the second half of collected tissue samples, half of them were processed for Masson dye staining. Tissue sections were viewed and photographed using a light microscope (Olympus, Japan). The collagen volume fraction (CVF), an index for the myocardial interstitial fibrosis, was averaged and calculated in the representative areas without the blood vessel and the endocardium in accordance with the previous report [36]. Percentage of CVF was calculated as the sum of all connective tissue areas divided by the sum of all connective tissues and muscle areas.

### Hematoxylin-eosin (HE) staining assessment

After IRI model and Apelin treatment, the hearts were rinsed off the blood, and subsequently immobilized for 48 h. The heart samples were dehydrated using different concentrations of ethanol and embedded in paraffin. The paraffin was cut into slices (5 μm) and stained with HE. IncuCyte™ S3 ZOOM cell imaging system (Essen BioScience, USA) was used to image the staining slices.

### Statistical analysis

All of the data in this study were analyzed with use of the SPSS software 13.0 (SPSS Inc., Chicago, Ull, USA). The data were described as mean ± standard deviation, which were gained from at least three independent experiments. The Student's t test and one-way ANOVA was used for the statistical analysis. NT-proBNP expression was presented by median (range) and analyzed by using Kruskal-Wallis. The P value less than 0.05 meant that the difference was significant.
